# Pulsed Electromagnetic Field (PEMF) stimulation as an adjunct to exercise: a brief review

**DOI:** 10.3389/fspor.2024.1471087

**Published:** 2024-09-09

**Authors:** Sheyda Ghanbari Ghoshchi, Maria Letizia Petroni, Alessandro Piras, Samuele Maria Marcora, Milena Raffi

**Affiliations:** ^1^ Department of Medical and Surgical Sciences, University of Bologna, Bologna, Italy; ^2^ Department of Quality of Life Studies, University of Bologna, Rimini, Italy; ^3^ Department of Biomedical and Neuromotor Sciences, University of Bologna, Bologna, Italy

**Keywords:** physical exercise, physical activity, PEMF, performance, recovery, sport, athletes, DOMS

## Abstract

Pulsed Electromagnetic Field (PEMF) therapy is a non-invasive treatment that utilizes electromagnetic fields to stimulate and promote natural healing processes within the body. PEMF therapy works by emitting low-frequency electromagnetic pulses, which penetrate deep into tissues and cells, enhancing cellular function and health. PEMF applications are vast, ranging from enhancing recovery in athletes to supporting overall well-being in everyday individuals. PEMF therapy is increasingly recognized in the realm of sports and physical activity for its profound benefits in enhancing performance, accelerating recovery, and preventing injuries. By improving circulation, enhancing tissue oxygenation, and promoting the body's natural healing processes, PEMF therapy has become an invaluable tool in sports medicine, contributing to optimized physical health and prolonged athletic careers. In this review, we explore the effects of PEMF on exercise and the underlying physiological mechanisms.

## 1 Introduction

Pulsed electromagnetic fields (PEMF) are a non-invasive medical therapy utilized in clinical treatments. The FDA granted approval for the application of PEMF in the repair of non-union fractures in humans in 1979 (1). PEMF therapy is currently employed for treating bone conditions like osteoporosis (2) and fractures (3). PEMF stimulation can be also used to improve tissue oxygenation, microcirculation and angiogenesis (4, 5). Kwan et al. (6) applied PEMF therapy on diabetic subjects, reporting an increase in microcirculation through the enhancement of capillary blood flow. Some authors have proposed PEMF stimulation as an adjunct to exercise (7, 8) but, to the best of our knowledge, there are no comprehensive articles examining the impact of PEMF on physical activity and sport. The aim of this review is to provide up-to-date summary of the current literature on PEMF and physical exercise, elucidating discrepancies, and identifying areas necessitating further research.

## 2 Biological rationale for PEMF stimulation as an adjunct to exercise

The application of external electromagnetic energy to an injured area triggers modifications in the cellular environment, promoting the restoration of tissue integrity and function (9–11). PEMFs have the potential to enhance tissue oxygenation, microcirculation, and angiogenesis in rats, human erythrocytes, and cell-free assays (4, 12, 13, 14). PEMF application has also showed modulatory effect on microvasculature and can result in its remodeling (15). Moreover, PEMF has the potential to resolve chronic inflammation via inducing changes in gene expression related to heme catabolism, removal of reactive oxygen species, and lipid mediator biosynthesis (16).

From the above-mentioned premises, we can state that the biological effects of PEMF stimulation may be useful to speed up recovery of specific muscles after exercises or to enhance the effects of exercise on specific body parts. Here we speculate about such practical applications.

### 2.1 Effects of PEMF on bones

The positive effects of PEMF stimulation on bone repair may be beneficial in the prevention and treatment of stress fractures in athletes and soldiers. Stress fractures occur primarily in the lower limbs and result from the repetitive mechanical overload associated with high-volume of endurance training. Standard treatment includes rest, ice and painkillers. Some painkillers may actually impair bone healing (17). PEMF stimulation may be used as an adjunct to exercise training in people at high risk of stress fractures to prevent their development. In athletes and soldiers recovering from stress fractures, PEMF stimulation may accelerate the return to sport or duty by enhancing the healing of the damaged bone tissue (18). Furthermore, PEMF stimulation may relieve pain in people with stress fractures due to its analgesic effects.

### 2.2 Analgesic effects of PEMF

Because pain is a significant barrier to exercise, the analgesic effects of PEMF stimulation may help people with osteoarthritis perform regular training to improve their physical function (19). A similar strategy may be applied in other situations in which musculoskeletal pain may limit the ability or willingness of people to perform exercise.

### 2.3 Effects of PEMF that may enhance recovery but may also reduce the hypertrophic response to resistance exercise

Exercise can induce significant muscle damage, especially when it includes eccentric muscle contractions (20). PEMF stimulation has many biological effects that may acutely enhance the recovery from such damage. These effects include reduced pain and inflammation, enhanced cellular repair and regeneration, stem cell activation and improved microcirculation, leading to better oxygenation and nutrient delivery to tissues (21). However, muscle damage and related inflammation have been proposed to be important stimuli for chronic muscle adaptations to resistance exercise (22). Therefore, it is important to directly investigate the net effect of PEMF stimulation on the muscle hypertrophy induced by resistance exercise.

### 2.4 Effects of PEMF stimulation on peripheral blood flow

PEMF stimulation is known to enhance peripheral blood flow. This effect may be particularly helpful as an adjunct to exercise in patients with impaired peripheral blood flow such as diabetic patients and people suffering from peripheral arterial disease. Exercise training is an important component in the management of these conditions (23, 24). PEMF stimulation may further enhance its beneficial effects in body parts particularly affected by vascular and microvascular alterations (25). Steward et al., (25) found that 12 weeks of PEMF stimulation let to improved endothelial vascular function and reduced blood pressure in hypertensive subjects showing that PEMF treatment could be a potential non-pharmacological and non-invasive strategy to manage vascular function and blood pressure in cohorts with peripheral vascular disease as well as hypertension.

## 3 Effect of PEMF on physical exercise

PEMF therapy impacts physical exercise with both acute and chronic effects. Acutely, PEMF can enhance muscle recovery by increasing blood flow and reducing inflammation, leading to immediate relief from muscle soreness and faster recovery times after workouts. Chronically, regular use of PEMF can improve overall muscular health, endurance, and performance by promoting cellular repair and optimizing metabolic function over time.

### 3.1 Effects of PEMF stimulation on the acute responses to exercise

Trofè et al. investigated the acute effects PEMF on muscle oxygenation during exercise. The authors found that PEMF enhanced muscle oxygenation and accelerated deoxyhemoglobin on-transition kinetics, indicating improved local oxygen extraction (7). In another study, the authors also found that PEMF stimulation increases the activity and metabolism of muscle fibers during physical exercise, therefore, PEMF has the potential to enhance muscular responses, particularly in low-intensity exercise scenarios (8).

Grote et al. aimed to assess the immediate and short-term effects of PEMF therapy on physiological parameters, particularly heart rate variability, following physical stress tests. Results showed that PEMF exposure influenced heart rate variability components related to sympathetically controlled blood flow rhythms, and accelerated recovery after physical strain. However, the application of PEMFs had no impact on participants overall well-being (26).

Viti et al. (27) investigated the effects of PEMFs stimulation using a PAP ion magnetic induction device, named PAPIMI, on autonomic nervous system activity in patients with chronic musculoskeletal pain. Results showed that PEMFs stimulation induced a significant parasympathetic response, which increased heart rate variability. Musculoskeletal pain conditions have been associated with a reduced heart rate variability [cfr (27)], thus, such acute autonomic change observed following PAPIMI intervention might be interpreted as a health status-related parasympathetic response. This study provided initial evidence on the potential physiological response induced by PEMF stimulation (PAPIMI device).

### 3.2 Effects of PEMF stimulation on the chronic responses to exercise

In a study by Parhampour et al., (28) PEMFs were applied with a 6-week resistance training program for patients with severe hemophilia A and osteoporosis. The aim was to enhance muscle strength, bone formation, and joint function. The authors enrolled 48 patients who were randomly assigned to one of four groups: resistance training alone, combined resistance training with PEMF, PEMF alone and control. Results showed that the absolute changes in the total score for joint function were significant for knees, ankles, and elbows in the resistance training alone and resistance training with PEMF, compared to the PEMF alone and control groups. The results indicated that combining PEMF stimulation with resistance training could be more effective than PEMF therapy alone.

Kandemir et al. (29) evaluated the effects of 3 months PEMF therapy in the treatment of subacromial impingement syndrome on 80 individuals randomly assigned to experimental (PEMF and exercise) or control (sham PEMF and exercise) groups. Both groups trained 5 days a week for a total of 20 sessions. Data were recorded before treatment (T0), after treatment (T1), and 12 weeks after the end of the intervention (T2). The results showed improvements in both groups compared with baseline. In the comparison between the two groups at T1 and T2, the PEMF group showed more improvements in most parameters.

### 3.3 Effects of PEMF on recovery after exercise

Jeon et al. explored the impact of PEMF therapy on recovery from delayed-onset muscle soreness (DOMS) after isometric exercise. A 10-min PEMF on the brachii biceps post-training improved DOMS symptoms in the following days, improving overall recovery quality. Additionally, PEMF treatment increased muscle activation frequency and reduced electromechanical delay, implying a shortened recovery time. However, no significant effect was observed on the peak of isometric force generation, warranting further studies to validate PEMF's positive influence on enhancing and expediting the recovery phase (30).

Galace de Freitas et al. aimed to assess the impact of PEMFs and exercises on pain reduction, function improvement, and muscle strength in patients with shoulder impingement syndrome. They reported that the combination of PEMF and shoulder exercises effectively enhanced function, muscle strength, and reduced pain in SIS patients. However, the results should be interpreted carefully due to the lack of significant differences between the groups (31).

## 4 Discussion

PEMF can lead to a better performance during exercise via several mechanisms.

The first mechanism by which PEMF can increase oxygenation is via increasing the tissue oxygenation through multiple ways including vessel diameter. Mayrovitz and Larsen (32) investigated the potential effects of a single 45-min pulsed radio frequency field treatment on peri-ulcer microcirculation in 15 subjects with diabetes and chronic foot or toe ulcers. Laser doppler measurements of red blood cell perfusion, volume, and velocity, along with skin temperature, were taken at both the peri-ulcer site and a contralateral nonulcerated limb site before and after treatment. They reported an increase in laser doppler perfusion at the peri-ulcer site, primarily attributed to an elevated volume component. No significant changes were observed at the contralateral control site or in skin temperature at either location. These results were further validated by Smith et al. (14) who showed a noticeable vasodilation in the cremasteric arterioles of PEMF treated rats. It has been suggested that PEMF exerts its vasodilatory effects via increasing the production of nitric oxide (NO). Bragin et al. (12) showed that 30 min of PEMF treatment induced cerebral arteriolar dilation leading to an increase in microvascular blood flow and tissue oxygenation that persisted for at least 3 h. The effects of PEMF were mediated by NO, given that the intravenous injection of the NO synthase inhibitor “L-NAME” prevented PEMF-induced changes in arteriolar diameter, microvascular perfusion, and tissue oxygenation. It has been suggested that the pathway by which PEMF increases NO production is increasing calcium influx. PEMF activates voltage-gated calcium channels which allows an influx of Ca^++^, consequently leading to the activation of NOS (11).

The second mechanism is via angiogenesis. Roland et al. (5) utilized a microsurgically created arterial loop model in a prospective randomized trial involving 108 rats (*n* = 12/group). The rats were exposed to pulsed magnetic energies of 0.1 and 2.0 gauss immediately postoperatively and for 4, 8, and 12 weeks. The results revealed a statistically significant increase in neovascularization in the treated animals compared to the control group (5).

The third mechanism regards oxygen consumption. PEMF can ameliorate oxygenation via increasing hemoglobin deoxygenation. Muehsam et al. (4) demonstrates that two clinically used electromagnetic field modalities, pulse-modulated radiofrequency and static magnetic field, independently increased the deoxygenation rate of human hemoglobin in a controlled cell-free assay. Trofè et al. examined the acute effects of PEMFs on muscle oxygenation and pulmonary oxygen kinetics during exercise in male cyclists using the previously mentioned data. Muscle oxygenation was improved by PEMF stimulation, as evidenced by higher primary and steady-state deoxyhemoglobin levels. Furthermore, compared to the non-stimulated condition, PEMF accelerated the kinetics of deoxyhemoglobin on-transition, resulting in a shorter time delay, time constant, and mean reaction time. The pulmonary oxygen kinetics and total oxygenation index did not change significantly during stimulation, despite the increasing lactate concentration. The results imply that without significantly altering overall oxygen kinetics during cycling, local PEMF stimulation can improve muscle oxygen extraction and use (7).

It is noteworthy that the impact of PEMF depends on the baseline characteristics of the individual. In the study of Grote et al. (26), 32 healthy male adults underwent standardized physical stress tests, followed by exposure to PEMFs of varying intensity. The research revealed that the influence of electromagnetic fields on the very low-frequency power spectral components of heart rate variability, reflecting sympathetically controlled blood flow rhythms, is contingent upon individual baseline factors. Notably, exposure to a specific magnetic field intensity accelerated recovery after physical strain compared to a placebo, and these effects quickly subsided upon cessation of magnetic field exposure (26).

### 4.1 Potential practical applications

Interestingly, the positive effects of PEMF therapy have been mostly reported in extreme situations, while outcomes for healthy adults remain yet to be elucidated (33). Athletes are frequently involved in preseason training camps where they undergo intensified training to optimize adaptations for the competition seasons (34). The initiation of such a training regimen after a period of inactivity poses a substantial physical challenge to athletes. Given the existing research and the widespread application of these devices in real-world scenarios, PEMF holds the potential to enhance athletic performance as athletes transition back to regular training seasons. This could be achieved by boosting recovery and reducing fatigue during training camps.

PEMF stimulation should thus gain attention in the athletic and therapeutic exercise communities for its potential benefits in enhancing performance, accelerating recovery, and promoting overall musculoskeletal health. PEMF application helps in reducing muscle soreness and stiffness after intense workouts, enabling athletes to recover faster and perform consistently at high levels (30). PEMF therapy is useful in managing both acute and chronic pain conditions enabling individuals undergoing rehabilitation to continue their therapeutic exercises without discomfort. The regular use of PEMF stimulation can improve muscle performance by enhancing cellular function and energy production leading to improved strength, endurance, and overall athletic performance (7, 8, 18, 29, 31). Athletes may experience better training outcomes and improved results in their competitive activities. Further, PEMF therapy is also effective in reducing inflammation and promoting the healing of joint and ligament injuries being a valuable tool for both injury prevention and recovery (18). Lastly, PEMF stimulation is a non-invasive, drug-free treatment option, which can be particularly important for athletes who wish to avoid the side effects of medications or the risks associated with invasive procedures.

PEMF stimulation offers a range of potential benefits for athletes and those involved in therapeutic exercise. From enhanced recovery and pain reduction to improved performance, PEMF therapy presents a promising adjunctive treatment to support physical health and optimize athletic outcomes.

### 4.2 Stimulation protocols

Although PEMF stimulation has been used for decades, there are currently no guidelines for categorizing PEMF. The main issue concerns the stimulation protocols, including time of exposure and frequency. However, those parameters are difficult to standardize. A discussion on the electromagnetic characteristics of PEMF stimulation can be found in the review by Flatscher et al. (21). Briefly, electromagnetic fields are composed of magnetic and electric fields that influence each other. PEMF applies intermittent, current pulse-generated magnetic field pulses over a short period of time. Tissues are affected by the applied PEMF field in two ways: firstly, the magnetic field creates a force on tissue-resided molecules which depend on their magnetic reactive properties, and secondly, the induced electrical field, which exerts a force on the ions present in the tissue (21); both result in a forced movement of ions or charged particles, such as proteins (35, 36). Mansourian and Shanei (37) performed a meta-analysis on PEMF *in vitro* studies showing that the effect of PEMF differs between cell type (stem cells) and origin (human/animal).

Almost every article discusses the heterogeneity of PEMF parameters used and positive effects are repeatedly reported A summary of results and characteristics of the reviewed research articles is shown in Table 1. Even though reliable research on such parameters has not been performed yet, PEMF is safe, non-invasive, relatively inexpensive and has been shown to positively contribute to many different conditions.

**Table 1:** PEMF characteristics and results of reviewed research articles.

Study	Mechanism	Result	PEMF characterists
Bragin et al. (12)	Tissue oxygenation	PEMF dilated cerebral arterioles, increased blood volume flow, enhanced capillary flow with an average increase in red blood cells flow velocity	The PEMF signal was a 27.12-MHz carrier modulated by a 3-ms burst repeating at 5 Hz
Ding et al. (18)	Bone formation	Early intervention with PEMF and parathyroid hormone resulted in the most pronounced protective effects among all the approaches against the bone stress injuries	The animals were exposed to various EMF waveforms, including the sinusoidal EMF (sin-EMF), single-pulsed EMF (sPEMF), or pulsed-burst EMF (PEMF) at the frequency of 15 or 50 Hz with various intensities (5, 20, or 50 G)
Fu et al. (9)	Bone formation	Proliferation and the osteogenic differentiation of human bone marrow stromal cells were increased	PEMF had frequency of 200 Hz and intensities of 0.6 and 1 T
Galace de Freitas et al. (31)	Exercise	Patients in the active PEMF group had a higher level of function and less pain at all follow-up time frames compared with baseline	The equipment used was a previously calibrated Magnetherp 330 pulsed with a frequency of 50 Hz and an intensity of 20 mT or 200 G
Grote et al. (26)	Recovery	PEMF accelerated recovery after physical strain	The mean magnetic pulse increase was 0.005, 0.03 and 0.09 T/s
Jeon et al. (30)	Recovery	The application of the PEMF was effective in reducing the physiological deficits associated with DOMS	The device generated time-varying PMF which has peak magnetic field intensity of 0.2 T and magnetic field pulse duration of 160 μs
Kandemir et al. (29)	Exercise	PEMF improved clinical symptoms	The PEMF was delivered by an electromagnetic field device on the painful shoulder area at 50 Hz frequency, 85 Gauss intensity
Kubat et al. (16)	Inflammation	PEMF treatment changed the relative amount of messenger (m)RNAs encoding enzymes involved in heme catabolism and removal of reactive oxygen species	The device emitted a 27.12 MHz radio frequency signal delivered in 42 µs pulses with a period of 1 KHz
Mayrovitz and Larsen (32)	Vascular	Increase in laser doppler perfusion at the peri-ulcer site, primarily attributed to an elevated volume component	PEMF frequency was 27.12 MHz with 600 pulse/s
Morris et al. (15)	Vascular	The application of chronic static magnetic field alters the adaptive microvascular remodeling response to mechanical injury	The tissue is exposed to a magnetic field gradient with a maximum of ∼20–60 mT/0.7 cm or 25–85 mT/cm
Muehsam et al. (4)	Tissue oxygenation	Exposure to static or pulsed magnetic fields increased the rate of deoxygenation	The signal consisted of a 27.12 MHz sinusoidal carrier configured to operate nonthermally through pulse modulation in 4 ms bursts, repeating at 5 Hz and peak magnetic field amplitude of 10 ± 1 µT
Parhampour et al. (28)	Exercise	The results indicated that combining PEMF stimulation with resistance training could be more effective than PEMF therapy alone	PEMF parameters: 30 Hz, 40 Gauss, and rectangular waveform
Kwan et al. (6)	Vascular	PEMF group showed major decrease in wound size and significant cumulative increase in cutaneous capillary blood velocity and increase in capillary diameter	Pulsed electromagnetic therapy was delivered at the frequency of 12 Hz, with magnetic flux density of 12 G
Roland et al. (5)	Vascular	The application of pulsed magnetic energies caused statistically significant increase in neovascularization	Pulsed magnetic energies of 0.1 and 2.0 gauss were applied using short pulses (2–20 ms) 27.12 MHz
Smith et al. (14)	Vascular	Local PEMF stimulation produced vasodilation in cremasteric arterioles in anesthetized rats	PEMF field strength measured in the apparatus was a positive amplitude of 18.8 T/s and a negative amplitude of 8.0 T/s
Stewart et al. (25)	Vascular	PEMF increased flow mediated dilation and reduced blood pressure	The device generated adjustable magnetic field strength range (*X*-axis: 0.22 ± 0.05 mT, *Y*-axis: 0.20 ± 0.05 mT, and *Z*-axis: 0.06 ± 0.02 mT) and working frequency (30 ± 3 Hz)
Trofè et al. (7)	Exercise	PEMF increased deoxyhemoglobin values, accelerated deoxyhemoglobin on-transition kinetic and increased lactate concentration	PEMF waveform consisted of a pulse-burst modulated 27.12 MHz sinusoidal carrier, with a 2 ms burst width repeated at 2 Hz, with peak magnetic field at the center of the loop 5 ± 1 µT
Trofè et al. (8)	Exercise	PEMF stimulation increased muscle activity in the warm-up condition and blood lactate concentration	PEMF waveform consisted of a pulse-burst modulated 27.12 MHz sinusoidal carrier, with a 2 ms burst width repeated at 2 Hz, with peak magnetic field at the center of the loop 5 ± 1 µT
Viti et al. (27)	Recovery and pain	Increased parasympathetic response, increased heart rate variability	Device with voltages reaching up to 40 kV and peak currents of several 10 kA, energy output per pulse of about 60 W (joules) with a magnetic induction of 50–150 mT

### 4.3 Conclusions and direction for future research

The data shown in this brief review demonstrate that PEMF application in physical activity and sport is a burgeoning field with considerable potential. Future research should focus on understanding how PEMF influences muscle metabolism, ATP production, and neuromuscular function. Detailed mechanistic studies are needed to elucidate how PEMF can be used to improve strength, endurance, and recovery at systemic level. Further studies should explore optimal frequencies, intensities, durations, and application timings relative to training sessions. Customizing protocols based on sport-specific demands and individual athlete profiles can enhance the practical utility of PEMF.

It is important to underline that, to date, the long-term effects of regular PEMF use on athletic performance and injury rates are underexplored. Longitudinal studies should track athletes over extended periods to assess how sustained PEMF use influences performance metrics, injury incidence, and career longevity providing insights into the viability of PEMF as a long-term tool in sports. Further, athletes of different ages and genders may respond differently to PEMF therapy. Future studies should investigate how factors such as age, sex, and hormonal variations influence the efficacy of PEMF enhancing personalized approaches to athlete care.

In conclusion, while the current literature on PEMF in physical activity and sport is promising, extensive research is necessary to fully harness its potential.
